# Pain Assessment Tool With Electrodermal Activity for Postoperative Patients: Method Validation Study

**DOI:** 10.2196/25258

**Published:** 2021-05-05

**Authors:** Seyed Amir Hossein Aqajari, Rui Cao, Emad Kasaeyan Naeini, Michael-David Calderon, Kai Zheng, Nikil Dutt, Pasi Liljeberg, Sanna Salanterä, Ariana M Nelson, Amir M Rahmani

**Affiliations:** 1 Department of Electrical Engineering and Computer Science University of California, Irvine Irvine, CA United States; 2 Department of Computer Science University of California, Irvine Irvine, CA United States; 3 Department of Anesthesiology and Perioperative Care University of California, Irvine Irvine, CA United States; 4 Department of Informatics University of California, Irvine Irvine, CA United States; 5 Department of Future Technology University of Turku Turku Finland; 6 Department of Nursing Science University of Turku Turku Finland; 7 Turku University Hospital University of Turku Turku Finland; 8 School of Medicine University of California, Irvine Irvine, CA United States; 9 School of Nursing University of California, Irvine Irvine, CA United States; 10 Institute for Future Health University of California, Irvine Irvine, CA United States

**Keywords:** pain assessment, recognition, health monitoring, wearable electronics, machine learning, electrodermal activity, post-op patients

## Abstract

**Background:**

Accurate, objective pain assessment is required in the health care domain and clinical settings for appropriate pain management. Automated, objective pain detection from physiological data in patients provides valuable information to hospital staff and caregivers to better manage pain, particularly for patients who are unable to self-report. Galvanic skin response (GSR) is one of the physiologic signals that refers to the changes in sweat gland activity, which can identify features of emotional states and anxiety induced by varying pain levels. This study used different statistical features extracted from GSR data collected from postoperative patients to detect their pain intensity. To the best of our knowledge, this is the first work building pain models using postoperative adult patients instead of healthy subjects.

**Objective:**

The goal of this study was to present an automatic pain assessment tool using GSR signals to predict different pain intensities in noncommunicative, postoperative patients.

**Methods:**

The study was designed to collect biomedical data from postoperative patients reporting moderate to high pain levels. We recruited 25 participants aged 23-89 years. First, a transcutaneous electrical nerve stimulation (TENS) unit was employed to obtain patients' baseline data. In the second part, the Empatica E4 wristband was worn by patients while they were performing low-intensity activities. Patient self-report based on the numeric rating scale (NRS) was used to record pain intensities that were correlated with objectively measured data. The labels were down-sampled from 11 pain levels to 5 different pain intensities, including the baseline. We used 2 different machine learning algorithms to construct the models. The mean decrease impurity method was used to find the top important features for pain prediction and improve the accuracy. We compared our results with a previously published research study to estimate the true performance of our models.

**Results:**

Four different binary classification models were constructed using each machine learning algorithm to classify the baseline and other pain intensities (Baseline [BL] vs Pain Level [PL] 1, BL vs PL2, BL vs PL3, and BL vs PL4). Our models achieved higher accuracy for the first 3 pain models than the BioVid paper approach despite the challenges in analyzing real patient data. For BL vs PL1, BL vs PL2, and BL vs PL4, the highest prediction accuracies were achieved when using a random forest classifier (86.0, 70.0, and 61.5, respectively). For BL vs PL3, we achieved an accuracy of 72.1 using a k-nearest-neighbor classifier.

**Conclusions:**

We are the first to propose and validate a pain assessment tool to predict different pain levels in real postoperative adult patients using GSR signals. We also exploited feature selection algorithms to find the top important features related to different pain intensities.

**International Registered Report Identifier (IRRID):**

RR2-10.2196/17783

## Introduction

Pain assessment is a key factor in successful pain management [1]. Inaccurate postoperative pain assessment may cause illnesses [2] and even long-term chronic issues [3]. Pain assessment tools for clinical use are in great demand. If communication ability is limited or even lost due to surgery or illness complications, it is difficult for a doctor to determine the patient's feelings. A proper pain assessment tool can offer an approximate pain level of that patient for further treatment. For now, the wide range of pain assessment methods still cannot determine the precise pain prevalence and levels for adults in hospitals [4]. That may cause incorrect treatment and lead to various problems and risks for patients. Painkillers have many side effects, and overtreatment of pain can trigger respiratory depression in the short term or substance use disorder in the long term [5]. However, undertreatment of pain may result in chronic pain, more health care costs, and physiological and psychological suffering [3,6]. All these issues mentioned are prevalent among noncommunicative patients [7]. A valid pain assessment tool would be truly transformative to health care delivery as clinicians could deliver pain treatments and assess response in real time. This would decrease unwanted side effects and recovery time from illness or a procedural intervention.

With the rapid development of Internet-of-Things (IoT) devices, including wearable devices, automated and continuous objective pain intensity assessment is possible [8]. The accuracy of these wearable devices has been evaluated in several studies. As an example, Mehrabadi et al [9] validated the accuracy of these devices in terms of sleep. Researchers try to identify nervous reaction to pain by monitoring the fluctuation in patients' physiological data, including electromyography, electrocardiography, photoplethysmography, and electrodermal activity (EDA) in real time [10,11]. Other research uses facial expression and head movement to accompany the physiological data [12,13]. These methods can quantify pain intensity, especially for poorly communicating patients [14]. However, all these methods to date use stimulated pain and were evaluated on healthy participants. Based on this observation, we developed the UCI iHurt Dataset (UCI_iHurtDB) [15]. The UCI_iHurtDB is the first multimodal dataset collected from postoperative adult patients suffering from real pain in hospitals. This dataset is planned to be released for research purposes in the near future.

Skin conductance or the EDA signal is considered a useful biomedical data point that corresponds to pain perception. Our skin produces sweat via over 3 million small tubular sweat glands. Sweat glands are distributed across the body but with the highest densities on the soles of the feet, palms and fingers, and forehead and cheeks. If a patient is exposed to a certain group of stimuli, they can be triggered to secrete moisture, termed emotional sweating. This results in a decrease in skin resistance, or in other words, an increase in skin conductance [16], which is also known as EDA or galvanic skin response (GSR). Other than pain, the EDA signal can extract a variety of valuable information from the human body. Rostami et al [17] highlighted an important insight that, by using the GSR signal, the biological impact of food on a person’s body can be captured.

Pain assessment research only using EDA data is limited. Eriksson et al [18] and Munsters et al [19] validated the relationship between EDA and pain for newborn infants, suitable for automated pain assessment due to their inability to communicate. By monitoring the EDA data during routine blood sampling or care intervention, they found EDA can differentiate between pain and no pain; however, more research is needed to achieve a clinical-grade level. Manivannan et al [20] verified whether the EDA could be used as a valid pain indicator for hypnotic analgesia with 10 participants. They used an iron disk to create mechanical pain in a laboratory setup. The experimental results show a clear relation between pain scores and EDA. None of these mentioned works used machine learning algorithms to create a classification model for pain assessment. Furthermore, their dataset includes healthy patients with various stimulus methods to cause pain. In another work, Susam et al [21] attempted to assess postoperative pain using EDA through a machine learning model. Their model could distinguish between clinical moderate-to-severe pain and no-pain conditions. However, their work only focused on children as a population.

To the best of our knowledge, for the first time in this paper, we present an automatic and versatile pain assessment tool to predict different pain levels in postoperative adults using only EDA signals as a physiological signal. In our pain assessment tool, we used 11 different time-domain features extracted from EDA signals for prediction. A feature selection algorithm is used to increase our tool's prediction accuracy and find the top-most important features related to pain intensity. To evaluate our results, we used different types of machine learning algorithms. Machine learning techniques and neural networks have been widely used in health monitoring domains. Zargari et al [22] used a combination of convolutional neural networks and recurrent neural networks to accurately track the position of in-mouth nutrient sensors. Mehrabadi et al [23] used convolutional neural networks to detect COVID-19 in patients with acute respiratory distress syndrome. These techniques are not limited only to health care domains. Ashrafiamiri et al [24] used deep neural networks to secure autonomous driving. To accurately validate our pain assessment algorithm's performance, we compared our results with the accuracy achieved for the pain models presented in [13]. Werner et al [13] used the BioVid Heat Pain database in their work in which participants were subjected to painful heat stimuli under controlled settings. This comparison aims to show that despite all the challenges in real hospital settings, our models can achieve comparable results. This also shows the feasibility of using artificial intelligence–based objective pain assessment for real patients.

## Methods

All methods of the study, including the data collection and pain assessment, were approved by the University of California Irvine Institutional Review Board (IRB, HS: 2017-3747). Potential candidates were screened for eligibility using the Acute Pain Service schedule and provided with a copy of the consent form to review for at least 24 hours before participation in research procedures.

### Study Description, Participants, and Recruitment

This study is a biomedical data collection study with postoperative patients reporting varying degrees of pain symptoms under local IRB approval supervision. We recruited 25 participants (age: 23-89 years) from the University of California, Irvine Medical Center. We recruited similar numbers of men and women (13 men and 12 women). We removed 3 participants' data from the final dataset due to data recording accidents such as excessive motion artifacts induced by hand movements, and 2 participants’ data were excluded since they were wearing the Empatica E4 watch on their IV arm, which resulted in unreliable EDA signal due to conditions such as skin rash and itching. The criteria for participant selection were as follows: (1) 18 years of age or older, (2) would receive a consult by the Acute Pain Service, (3) no barriers to communication, (4) able to provide written informed consent, and (5) have intact and healthy facial skin. Participants were excluded if they had any of the following: (1) any diagnosed condition affecting cognitive function like dementia or psychosis; (2) any diagnosed condition affecting the central nervous system, facial nerves, or muscles; (3) deformities on the hand or other parts of the body that prevent sensor placement; and (4) significant facial hair growth in the area for sensor attachment. Patients were selected if they satisfied the inclusion and exclusion criteria and determined to be enrolled in this study voluntarily. Verbal and written consent was acquired before initiation of the study.

### Study Design

After the recruitment procedure, GSR data were collected for approximately 30 minutes continuously from the patients in their private room. We separated these 30 minutes of data monitoring into 2 phases. In the first phase, an artificial pain generator called a transcutaneous electrical nerve stimulation (TENS) [25] unit was used to let participants have an initial impression of multiple pain levels and let researchers obtain baseline biosignals from the person. The TENS unit stimulates different levels of acute pain by delivering small electrical impulses through electrodes attached to the participant's skin with adhesive pads. Participants were told to gradually increase the TENS unit's intensity to a tolerable level for them and then hold it for at least 10 seconds. After this, we decreased the intensity back to level 0. In the second phase, participants engaged in low-intensity activities such as walking, coughing, sitting up, or lifting legs that caused an expected degree of pain. To improve data reliability for the following analysis, the entire data monitoring process was repeated sequentially. The monitored person's self-report of pain was measured using the Numeric Rating Scale (NRS), a segmented numeric version of the Visual Analogue Scale (VAS). The VAS is a validated, subjective measure for acute and chronic pain. Pain scores are recorded by making a mark on a 10-cm line representing a sequence between “no pain” and “worst pain.” NRS quantifies the pain intensity to 10 levels (0 is no pain, and integers 1 to 10 represent different pain levels, with 10 being the highest pain imaginable) [26,27].

### Data Collection

We used the Empatica E4, the commercially available wristband, to monitor the EDA data. The wristband is simple to position, and participants can maneuver easily without the device impeding their movements in any way. The wristband's internal memory allows recording up to 36 hours of data and wireless data transmission. The E4 wristband is rechargeable, with a charging time of fewer than 2 hours. An EDA sensor is embedded in the E4 wristband. This sensor measures the fluctuating changes in certain electrical properties of the skin. 

### GSR Feature Extraction Pipeline Architecture

**Figure 1:**
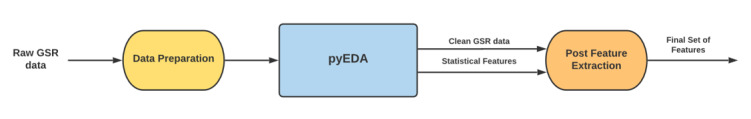
Galvanic skin response (GSR) feature extraction pipeline. EDA: electrodermal activity.

Figure 1 shows our pipeline architecture for preparing the data and extracting the set of features for classification. There are 3 different sections in this pipeline: (1) Data Preparation, (2) pyEDA [28], (3) Post Feature Extraction.

#### Data Preparation

The primary purpose of the Data Preparation in our pipeline is to synchronize the data with the labels. To prepare the data for feature extraction, we extracted the original signals’ slices that match with their corresponding labels. With this aim, the slices of GSR data and their labels are collected in this part to be fed to the pyEDA for pre-processing and feature extraction.

#### pyEDA

**Figure 2:**
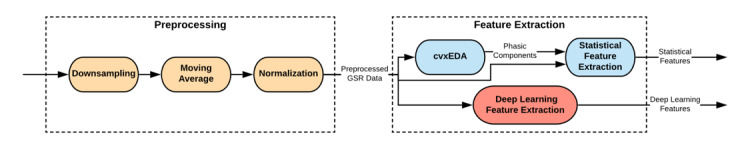
Pipeline architecture of the pyEDA. EDA: electrodermal activity; GSR: galvanic skin response.

The architecture of the pyEDA is shown in Figure 2. According to this figure, Preprocessing and Feature Extraction are the 2 main stages in this pipeline.

In the preprocessing stage of the pyEDA pipeline, at first, the data are down-sampled; then, a moving average is used to smooth the data and reduce the artifacts such as body gestures and movements. In the end, the data are normalized to become suitable for classification models.

If the GSR data are collected at 128 Hz, it can safely be down-sampled to a 20 Hz sampling rate. This down-sampling has been done to conserve memory and processing time of the data. In this work, we did not down-sample the data since the original data are already sampled at 4 Hz, which is good in terms of time and memory usage.

In this work, several steps were taken to remove motion artifacts from the GSR signal. First, we used a moving average across a 1-second window to remove the motion artifacts and smooth the data. Second, a low-pass Butterworth filter on the phasic data was applied to remove the line noise. Lastly, preprocessed GSR signals corresponding to each different pain level were visualized to ensure the validity of the signals.

The pyEDA uses 2 different algorithms for feature extraction (Statistical Feature Extraction and Deep Learning Feature Extraction). The parameters of the Deep Learning Feature Extraction part of the pipeline are set and tuned for stress detection; therefore, in this work, we only used the features extracted by the Statistical Feature Extraction algorithm.

The number of peaks, the mean, and the max peak amplitude are the 3 different statistical features that are extracted in the pyEDA. The GSR signals consist of 2 main components: skin conductance level, also known as the tonic level of GSR, and skin conductance response, also called the phasic component of GSR. The GSR peaks or bursts are considered the variations in the phasic component of the signal. Therefore, the most important part in extracting the peaks of the GSR signal is to extract its phasic component. Based on Figure 2, the pyEDA tool uses the cvxEDA algorithm [29] to extract the phasic component. Then, the phasic component and the preprocessed GSR data are fed to the Statistical Feature Extraction module to extract the 3 mentioned features (number of peaks, mean GSR, and max peak amplitude).

#### Post Feature Extraction

We also extracted the features that were used in the work by Werner et al [13] for the GSR signals. The preprocessed GSR signals and the set of features (number of peaks, mean GSR, and max peak amplitude) were fed into the Post Feature Extraction module to extract these features.

The maximum value of the peaks, range, standard deviation, interquartile range, root mean square, mean value of local maxima, mean value of local minima, mean of the absolute values of the first differences, and mean of the absolute values of the second differences are the extra features that were extracted in this part. Table 1 shows all the extracted features with their descriptions.

The mean of the absolute values of the first differences (mavfd) is calculated as:

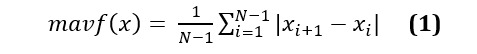



The mean of the absolute values of the second differences (mavsd) is calculated as:

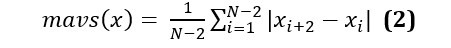



**Table 1:** Extracted galvanic skin response (GSR) features with their descriptions.

Feature	Description
Number of peaks	The number of peaks
Mean	The mean value of the signal
Max	The maximum value of the peaks
Range	The difference between the maximum and the minimum value of the signal
STD	Standard deviation of the signal
IQR	The difference between upper and lower quartiles of the signal
RMS	Root mean square of the signal
Mean minima	The mean value of local minima of the signal
Mean maxima	The mean value of local maxima of the signal
mavfd	The mean of the absolute values of the first differences
mavsd	The mean of the absolute values of the second differences

### Classification

#### Feature Selection

One of the key components in machine learning is to select the set of features that has the highest importance in classification. Performing feature selection on the data reduces overfitting, reduces training time, and improves accuracy. By removing the set of features that are not informative for our classification and only add complexity to our model, there is less opportunity to make decisions based on noise, making the model less over-fitted. Fewer data means less training time. In the end, by having more informative data and fewer misleading data, the accuracy of the model increases.

Random forests [30] are among the most popular machine learning methods. They provide 2 methods for feature selection: mean decrease impurity and mean decrease accuracy. In this work, we used a mean decrease impurity method for feature selection.

Mean decrease impurity is also sometimes called Gini importance. Random forest is an ensemble learning algorithm consisting of several decision trees. The decision tree is a tree-like model of decisions in which every node is a condition on one of the features. These nodes separate the data into 2 different sets so that in the optimal scenario, the data with the same labels end up in the same set. Impurity is the measure based on which the optimal condition is chosen on every node. Mean decrease impurity for each feature is defined as the total decrease in node impurity averaged over all ensemble trees. The features are ranked according to this measure.

#### Labeling the Features

Table 2 shows the distribution of labels in the UCI_iHurtDB. There exists 11 different pain levels in this dataset. It is noticeable that the distribution of different pain levels for the patients is imbalanced (4 occurrences of pain level 10, but 83 occurrences of pain level 4, as an example). This is understandable due to the subjective nature and the different sources of pain among our patients.

**Table 2:** Distribution of labels in the UCI_iHurtDB before down-sampling.

Pain level	Frequency, n
PL0	37
PL1	52
PL2	37
PL3	61
PL4	83
PL5	44
PL6	32
PL7	16
PL8	46
PL9	26
PL10	4

In the work by Werner et al [13], there were 5 different pain levels, including the baseline level. To properly compare our pain assessment algorithm with their work, we down-sampled our 11 classes to 5 classes. The key factor in this down-sampling is to ensure that the distribution of the labels is as balanced as possible. As a result, we considered pain levels 1-3 as new pain level 1 (PL1), pain level 4 as new pain level 2 (PL2), pain levels 5-7 as new pain level 3 (PL3), and pain levels 8-10 as new pain level 4 (PL4). Based on Table 2, there are only 37 data points for the baseline. To increase the number of samples for the baseline to make our labels more balanced, we up-sampled PL0 based on the reported PL0 data by the patients. We ensured these new baseline data were close enough to the reported pain level 0 labels (less than 10 seconds difference) and had no overlap with other labels. These assumptions were made to make sure (1) we were not reproducing any data and (2) the patients had the same pain level 0 for these new timestamps. By doing this procedure for all the participants, our number of samples for pain level 0 increased from 37 to 86.

Table 3 shows the distribution of the down-sampled labels and the new baseline. The distribution of the new labels is appropriately balanced. Still, for PL1, the number of samples is slightly higher than the rest of the classes. This is because we down-sampled our pain levels to 4 different classes to make our settings comparable with the work by Werner et al [13].

**Table 3:** Distribution of labels in the UCI_iHurtDB after down-sampling.

Pain level	Frequency, n
PL0	86
PL1	150
PL2	83
PL3	92
PL4	76

#### Machine Learning Algorithms

We used machine learning–based algorithms to evaluate the performance of our pain assessment algorithm. Two different classification methods were used here: (1) k-nearest-neighbor with *k* between 1 and 20 and (2) random forest with a depth between 1 and 10. The k-nearest-neighbor method uses *k* number of nearest data points and predicts the result based on a majority vote [31]. The random forest classifier is an ensemble learning algorithm that fits several decision tree classifiers on various subsamples of the dataset and uses averaging to improve the predictive accuracy and control over-fitting [30]. We used the Scikit-learn library to create our classification models [32]. Scikit-learn is a free software machine learning library for the Python programming language. It features various classification, regression, and clustering algorithms, including k-nearest-neighbor and random forest.

To accurately evaluate the performance of our classification models, we used a cross-validation method [33]. Cross-validation is one of the most popular algorithms used to truly estimate a machine learning model's accuracy on unseen data. It achieves this by training a model using different subsets of data and obtaining the average accuracy on the rest of the data as a test. In this work, we used leave-one-out cross-validation to evaluate our result. We considered all the data acquired from one of the patients as a test and created our pain model using the rest of the patients. We repeated this procedure for each patient as a test. Each time, we created our pain model from scratch without considering the current test patient data or any information from the previous pain models. The final accuracy of the model was obtained by averaging the accuracy of all constructed pain models.

## Results

A total of 25 patients with acute pain were engaged by the Acute Pain Service and recruited for this study. Of these 25 participants, 5 were removed from our study due to problems in the data collection process due to rapid hand movements or unreliable EDA signal due to conditions such as skin rash and itching resulting from wearing the Empatica E4 watch on their IV arm. The average age of patients in this study was 54.45 (SD 17.44, range 23-89) years; 55% (11/20) of the patients were male, and 45% (9/20) of patients were female. All patients were taking the standard-of-care postoperative pain medications at the time of the study. Enrolled participants agreed to perform the research protocol for a median of 4 (IQR 3-6) days after surgical intervention. The nature of the procedures for each participant included the following domains: 45% (9/20) general surgery (diagnostic laparoscopy, exploratory laparotomy, and vascular), 15% (3/20) trauma (thoracic pain and rib plating), and 10% (2/20) urology (cystectomy and bladder augmentation). Also, 40% (8/20) of enrolled participants received standard-of-care epidural analgesia provided by the acute pain service team at the time of research participation. The remaining participants were receiving oral and intravenous analgesics for pain control (Table 4).

**Table 4:** Summary of patients’ demographic characteristics for this study including exclusions.

Variable		Value	Range
Patients excluded for hand movement noise, n (%)		3 (12)	N/A^a^
Patients excluded for IV arm effect, n (%)		2 (8)	N/A
Age (years), mean (SD)		54.45 (17.44)	23-89
Gender, male, n (%)		11 (55)	N/A
Weight (kg), mean (SD)		75.24 (14.60)	52.2-102
Height (cm), mean (SD)		170.07 (9.00)	154.9-185.9
BMI (kg/m^2^), mean (SD)		26.21 (5.75)	15.1-38.6
**Nature of the procedure, n (%)**			
	General surgery	9 (45)	N/A
	Orthopedics	6 (30)	N/A
	Trauma	3 (15)	N/A
	Urology	2 (10)	N/A

^a^N/A: not applicable.

To show that our pain assessment algorithm can achieve comparable results to the work by Werner et al [13], we used identical settings as their work. Werner et al [13] used 5 different pain levels, including the baseline. They also considered 5.5-second windows for the GSR data. Therefore, in the Data Preparation part of our pipeline, we considered 5.5-second windows for the slices of the GSR data for feature extraction (2.75 seconds before and after each timestamp). Furthermore, as discussed in the Methods section, we down-sampled the pain levels from 11 classes to 5 classes to make them similar with their labels.

At first, we used the set of features that was used in the work by Werner et al [13] for classification without any feature selection. The maximum value of the peaks, range, standard deviation, interquartile range, root mean square, mean value of local maxima, mean value of local minima, mean of the absolute values of the first differences, and mean of the absolute values of the second differences are the features that were used here.

We used leave-one-person-out cross-validation using k-nearest-neighbor and random forest algorithms. We reported the accuracy based on 4 different pain models (BL vs PL1, BL vs PL2, BL vs PL3, and BL vs PL4). Table 5 shows the comparison of the validation accuracy achieved by our classifiers with that by the pain models of Werner et al [13].

**Table 5:** The validation accuracies in comparison with Werner et al [13] using the same set of features.

Binary classification	RF^a^	KNN^b^	Werner et al [13]
BL vs PL1	84.0	74.4	55.4
BL vs PL2	66.3	67.5	60.2
BL vs PL3	57.2	65.0	65.9
BL vs PL4	55.2	53.0	73.8

^a^RF: random forest.

^b^KNN: k-nearest-neighbor.

According to these data, for the first 2 pain models (BL vs PL1 and BL vs PL2), we achieved a higher accuracy using both of our classifiers in comparison with Werner et al [13]. For the third pain model, our accuracy is also close to their models, with less than 1% difference using the k-nearest-neighbor classifier. For the fourth model, the accuracy of our models was noticeably lower than their models. As the next step, we added 2 more features (the number of peaks and the mean of the GSR data) to our set of features and then selected the most important ones using the mean decrease impurity method to improve the accuracy.

To obtain the best set of classification features, we ran leave-one-person-out cross-validation on different pain models using random forest classifiers. We computed the Gini importance of the features on the training data and selected the top *k* number of features for training the model and classification (2-11 were considered to be possible values for *k*). Since we had a different number of folds, we could have different sets of features for each fold. We considered the set of features that was used in most of the folds as the final set of features for the current pain model. Table 6 shows the selected features for each pain model. Descriptions for each of these features can be found in Table 1.

**Table 6:** Selected set of features for each pain model.

Pain models	Set of features
BL vs PL1	Mean, max, RMS^a^, and mean maxima
BL vs PL2	Mean, max, RMS, and mean maxima
BL vs PL3	Max and RMS
BL vs PL4	Mean, max, IQR, RMS, and mean maxima

^a^RMS: root mean square.

According to this table, the maximum value and root mean square of the signal are 2 features that were selected in all the pain models. The mean value of the local maxima and the mean value of the signal were also selected for all the pain models except the third one. The difference between upper and lower quartiles of the signal (IQR) is a feature that was selected as an important feature for classification only for the BL vs PL4 pain model.

After the set of features for each pain model were obtained, we ran one-person-leave-out cross-validation for k-nearest neighbor and random forest algorithms using these sets of new selected features to achieve the final results.

Table 7 shows the validation accuracy comparison of our models with those by Werner et al [13] using feature selection for 5.5-second windows. As shown in this table, by using feature selection, our classifiers' accuracy improved compared to those shown in Table 5. For all the pain models except the fourth one, we were able to achieve higher accuracy than the pain models by Werner et al [13]. For BL vs PL4, our accuracy was still about 10% less than their work. In the Discussion section, we explain the potential reasons for this difference in our model.

**Table 7:** Validation accuracies in comparison with the work by Werner et al [13] using feature selection.

Binary classification	RF^a^	KNN^b^	Werner et al [13]
BL vs PL1	86.0	76.8	55.4
BL vs PL2	70.0	69.1	60.2
BL vs PL3	69.8	72.1	65.9
BL vs PL4	61.5	60.0	73.8

^a^RF: random forest.

^b^KNN: k-nearest-neighbor.

## Discussion

### Strengths

We are the first to develop an automatic and versatile pain assessment tool using GSR signals to accurately predict different pain intensities in postoperative adult patients. According to our results, using identical settings and even the exact GSR features used in the work by Werner et al [13], we can achieve higher accuracy in 2 of the 4 different pain models (BL vs PL1 and BL vs PL2). Also, for the third pain model (BL vs PL3), using the k-nearest-neighbor classifier, we can achieve the same level of accuracy with less than 1% difference. Machine learning algorithms and feature selection are not a part of the pain assessment algorithm settings. As a result, to show the true strength of our pain assessment algorithm, first, we added 2 more features to our set of features (the number of peaks and the mean value of the signal). Then, we used the mean decrease impurity method to select the most important features for classification. According to Table 7, we can achieve higher accuracy for the first 3 pain models using this procedure. Based on our results, for the first pain model (BL vs PL1), our accuracy is considerably higher than the accuracy achieved by Werner et al [13] (with and without feature selection). By feature selection, we are using a much lower number of features in our pain models. This reduces the complexity of our pain models. 

Furthermore, we present the most important features for each classification model. According to Table 5, the maximum value and root mean square of the signal appear to be the 2 most important features for pain classifications for postoperative adult patients. The mean value and mean value of the local maxima of the signal are considered to be the next 2 important features for classification.

Our results show that, in addition to healthy participants from previous studies, we can detect different intensities of pain using only GSR data in real-life patients in the hospital. GSR data can easily be collected using affordable wearable devices such as the Empatica E4 used in our study. Therefore, our pain assessment algorithm is really beneficial in providing valuable information to hospital staff and caregivers to better manage pain, especially for those patients who cannot communicate.

### Limitations

As our work's limitations, we used data collected from postoperative adult participants in our pain assessment algorithm. Data collection in real life may have led to more motion artifact noise in our physiological signals than data collection in a lab setting (especially for GSR data collected from Empatica E4 connected to their wrist). The motion artifacts were stronger for higher pain levels since the patient was in more unbearable pain. 

Another limitation in our work can be the lack of balanced pain levels for all the patients. Since our data were collected from real postoperative adult participants, it is possible that the patients in the experiment did not experience and report all different pain levels. This limitation could be more noticeable at higher pain levels.

Based on our results, we were not able to achieve the accuracy of that by Werner et al [13] for BL vs PL4 (with a 10% difference). The limitations mentioned in previous paragraphs can explain the potential reasons behind this difference. 

Furthermore, we could not find a significant difference between different pain levels in our study. We believe this is because the variations in GSR signal response to different pain levels are more alike to be distinguished easily. It is worth mentioning that most state-of-the-art pain assessments mainly focus on comparing baseline with other pain levels (eg, Werner et al [13], if the patient is in pain or not).

According to our results, the accuracy of our pain models generally decreases with increasing pain levels. This is the opposite of the models from Werner et al [13]. We believe this is due to our work's limitations mentioned in the earlier paragraph (motion artifact in signals and lack of balanced pain levels for all the patients). Both of these mentioned limitations could be more noticeable at higher pain levels. Therefore, it is understandable that in this work, where the data were collected from postoperative patients in real life, our models' accuracy decreased with increasing pain levels.

### Conclusions

In conclusion, according to our results, we can evaluate the performance of our pain assessment algorithm. This evaluation shows that it is feasible to predict different pain levels in real postoperative adult participants using only the EDA data. Furthermore, we showed that the mean value, maximum value, root mean square, and mean value of the local maxima of the signal are the most important features for pain classification of real patients in pain. Multimodel pain assessment methods can be implemented as future work to increase our pain assessment models' performance.

## Abbreviations

EDA: electrodermal activity
GSR: galvanic skin response
IoT: Internet of Things
IRB: institutional review board
NRS: Numeric Rating Scale
TENS: transcutaneous electrical nerve stimulation
VAS: Visual Analogue Scale
